# Impact of temperature and humidity on performance of the fecal immunochemical test for advanced colorectal neoplasia

**DOI:** 10.1038/s41598-019-44490-y

**Published:** 2019-07-08

**Authors:** Chan Hyuk Park, Yoon Suk Jung, Nam Hee Kim, Mi Yeon Lee, Jung Ho Park, Dong Il Park, Chong Il Sohn

**Affiliations:** 1 Department of Internal Medicine, Hanyang University Guri Hospital, Hanyang University College of Medicine, Guri, Korea; 2 Division of Gastroenterology, Department of Internal Medicine, Kangbuk Samsung Hospital, Sungkyunkwan University School of Medicine, Seoul, Korea; 3 Preventive Healthcare Center, Kangbuk Samsung Hospital, Sungkyunkwan University School of Medicine, Seoul, Korea; 4 Division of Biostatistics, Department of R&D Management, Kangbuk Samsung Hospital, Sungkyunkwan University School of Medicine, Seoul, Republic of Korea; 5 Division of Gastroenterology, Department of Internal Medicine, Kangbuk Samsung Hospital, Sungkyunkwan University School of Medicine, Seoul, Korea; 6 Division of Gastroenterology, Department of Internal Medicine, Kangbuk Samsung Hospital, Sungkyunkwan University School of Medicine, Seoul, Korea; 7 Division of Gastroenterology, Department of Internal Medicine, Kangbuk Samsung Hospital, Sungkyunkwan University School of Medicine, Seoul, Korea

**Keywords:** Colonoscopy, Colorectal cancer

## Abstract

Although it is known that ambient temperature can affect the diagnostic performance of the fecal immunochemical test (FIT), the impact of other weather parameters, including humidity, on the sensitivity of FIT remains to be further investigated. We aimed to evaluate the impact of ambient temperature and humidity on the performance of FIT for screening for advanced colorectal neoplasia (ACRN). We included asymptomatic individuals who had undergone both screening colonoscopy and FIT. The diagnostic performance of FIT, including its sensitivity, was analyzed according to the ambient temperature and humidity on the day that FIT was performed. Temperature and humidity were divided into five levels. Among 35,461 participants, 589 (1.7%) had ACRN. The positivity rate of FIT was lower at ≥24 °C (3.1%) than at <0 °C (3.9%), 0–8 °C (4.3%), and 8–16 °C (3.9%). It was also lower at 80–90% humidity (3.1%) than at < 60% humidity (3.9%). Multivariable analysis showed that high ambient temperature (≥24 °C) with high ambient humidity (≥80%) was associated with a low positivity rate of FIT (odds ratio [OR] 0.62, 95% confidence interval [CI] 0.44–0.86). Sensitivity tended to decrease at high ambient temperature (<24 °C vs. ≥24 °C; 20.8% vs. 14.6%, *P* = 0.110) and was significantly lower at high ambient humidity (<80% vs. ≥80%; 21.0% vs. 12.5%, *P* = 0.044). The multivariable analysis also showed that high ambient humidity was independently associated with low sensitivity of FIT (OR 0.54, 95% CI 0.28–0.96). In conclusion, high ambient humidity decreased the sensitivity, while high ambient temperature along with high ambient humidity decreased the positivity rate of FIT.

## Introduction

Colorectal cancer (CRC) is the third most common cancer and the fourth leading cause of cancer-related mortality in the world^1^. The US Preventive Services Task Force recommends screening for CRC starting at 50 years of age and continuing until age 75^2^. In addition, the US Multi-Society Task Force strongly recommends colonoscopy every 10 years or an annual fecal immunochemical test (FIT) as first-tier options for screening individuals at average risk of colorectal neoplasia (CRN)^3^. Although colonoscopy can effectively prevent CRC through resection of colorectal adenoma, colonoscopic screening has not been popular worldwide owing to limited medical resources^4,5^. FIT is now regarded as the best noninvasive tool for CRC screening^6^. The Asia Pacific guideline indicates FIT as the first choice for CRC screening in resource-limited countries^7^. FIT has been adopted as a population-based screening program in many countries, including Korea^4,5,8^.

It is well known that FIT is superior to the guaiac test in terms of efficacy in preventing CRC, through enhanced detection of advanced adenomas^9–11^. The sensitivity and specificity of FIT are maintained across individuals with different clinical risk factors^12^. However, there is concern that the performance of FIT is influenced by seasonal variations. Several studies from different countries, including Korea, France, Italy, and Netherlands, reported that the positivity rate of FIT is lower in summer^13–16^. High ambient temperature is suggested as a possible factor that lowers the positivity rate of FIT^14,17^. This is an important issue, because the exposure of fecal samples to a high ambient temperature may be unavoidable in population-based screening^13^.

However, the impact of high ambient temperature on the performance of FIT is yet to be further investigated. The positivity rate of FIT depends on the prevalence of CRC or advanced CRN (ACRN); therefore, it is better to evaluate the performance of FIT in terms of sensitivity and specificity rather than its positivity rate. In most studies, however, sensitivity and specificity could not be assessed because only participants who showed positive results for FIT were offered colonoscopy^14,15^. In addition, a contradictory study shows that ambient temperature alone does not affect the performance of FIT^18^. Not only ambient temperature but also other weather parameters, such as humidity, may affect seasonal variation in the performance of FIT. In an experimental study, high humidity was associated with an increased rate of hemoglobin degradation^19^. In this study, we aimed to evaluate the impact of weather parameters, including ambient humidity as well as ambient temperature, on the performance of FIT using a large cohort of asymptomatic individuals who underwent both colonoscopy and FIT. The positivity rate, sensitivity, and specificity of FIT for ACRN were assessed according to ambient temperature and humidity.

## Results

### Baseline characteristics of participants

The medical records of 46,654 participants who underwent both screening colonoscopy and FIT were reviewed. Of these participants, 5,544 were excluded because they had previously undergone a colonic examination, colorectal surgery, or surgery for CRN. In addition, 74 participants with inflammatory bowel disease were excluded. Of the remaining 41,036 participants, 3,171 were excluded due to poor bowel preparation and 2,404 because of incomplete data. A final total of 35,461 participants was included in this study.

Table 1 shows participants’ characteristics at baseline and the timing of FIT. The age of participants ranged from 30 to 85 years. The prevalence of CRN, ACRN, and CRC was 16.7%, 1.7%, and 0.08%, respectively. Five participants with ACRN had both CRC and advanced adenoma.

**Table 1:** Baseline patient characteristics, and timing of fecal immunochemical test.

Variable	Total
N	35461
Age, year, mean ± SD (range)	43.0 ± 8.6
Male, n (%)	25366 (71.5)
Family history of CRC, n (%)	1302 (3.7)
**Smoking history, n (%)**	
Never smoker	17815 (50.2)
Former smoker	8765 (24.7)
Current smoker	8881 (25.0)
Obesity (BMI ≥ 25 kg/m^2^), n (%)	12644 (35.7)
Hypertension, n (%)	3098 (8.7)
Diabetes, n (%)^a^	1922 (5.4)
Old cerebrovascular accident, n (%)	158 (0.4)
Dyslipidemia, n (%)	4427 (12.5)
Use of NSAIDs, n (%)	1347 (3.8)
CRN, n (%)	5936 (16.7)
ACRN, n (%)^b^	589 (1.7)
Advanced adenoma	567 (1.6)
Cancer	27 (0.08)
**Quarter of FIT, n (%)**	
1st quarter (January - March)	5350 (15.1)
2nd quarter (April - June)	9505 (26.8)
3rd quarter (July - September)	11544 (32.6)
4th quarter (October - December)	9062 (25.6)
**FIT device**	
HM-JACK test	9864 (27.8)
OC-SENSOR DIANA test	25597 (72.2)

^a^There are missing values in 10 individuals.

^b^Five participants with ACRN had both CRC and advanced adenoma.

CRC, colorectal cancer; BMI, body mass index; NSAID, nonsteroidal anti-inflammatory drug; CRN, colorectal neoplasia; ACRN, advanced colorectal neoplasia; FIT, fecal immunochemical test; SD, standard deviation; NA, not applicable.

### Weather information during the study period

**Figure 1:**
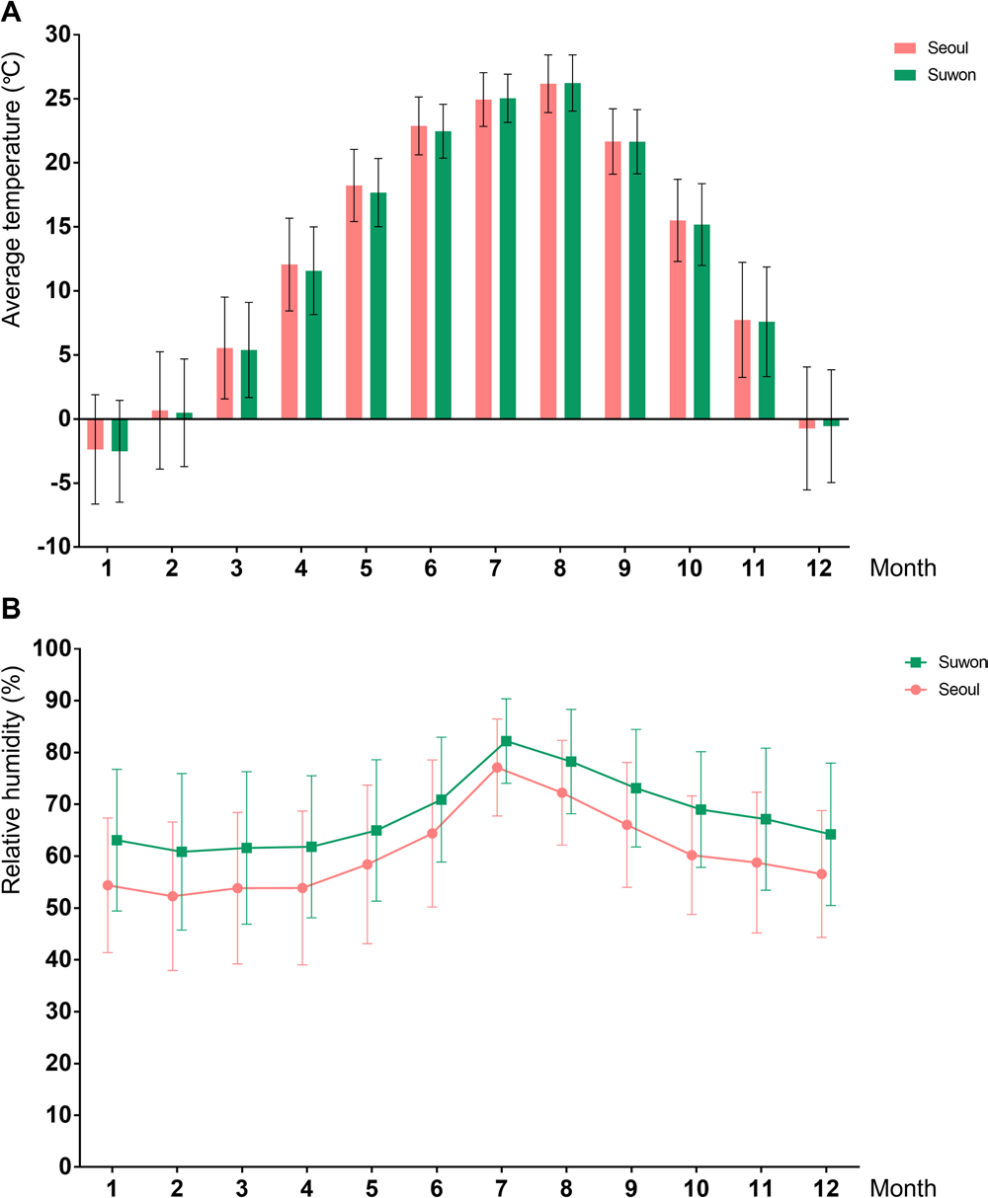
Monthly average outside temperatures (**A**) and humidity (**B**) from 2004 to 2015 in Seoul and Suwon in Korea, where the health examination centers in this study are located. Bar represents 95% confidence interval.

The monthly average temperature and relative humidity during the study period (from 2004 to 2015) are shown in Fig. 1. Average temperatures through the year were similar in the two regions (Seoul and Suwon). The average temperature was the highest in August, July, and June in both Seoul and Suwon (26.2 °C, 24.9 °C, and 22.9 °C, respectively, in Seoul, and 26.2 °C, 25.0 °C, and 22.5 °C, respectively, in Suwon). Changes in relative humidity through the year showed a similar pattern in the two regions, although the humidity in Suwon tended to be higher than in Seoul. Relative humidity was the highest in July, August, and September in both Seoul and Suwon (77.1%, 72.2%, and 66.0%, respectively, in Seoul, and 82.2%, 78.3%, and 73.1%, respectively, in Suwon).

### Performance of fecal immunochemical test according to quarter

**Figure 2:**
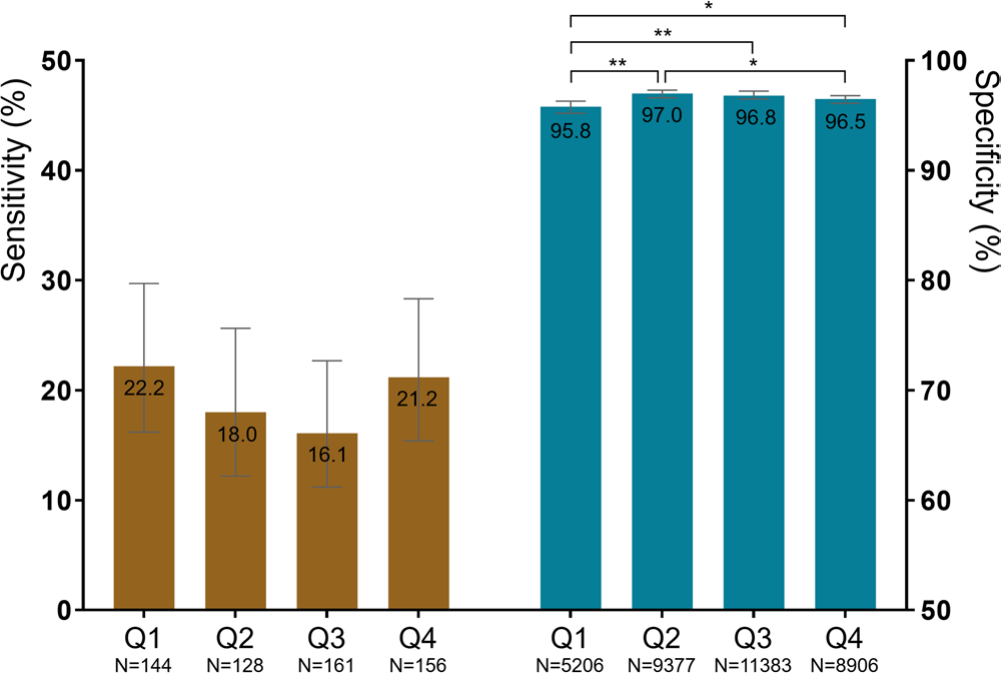
Diagnostic performance of fecal immunochemical test for advanced colorectal neoplasia. Left and right panels indicate the sensitivity and specificity, respectively. Q1, Q2, Q3, and Q4 indicate the first, second, third, and fourth quarters, respectively. Bar represents 95% confidence interval. *P < 0.05, **P < 0.01.

Figure 2 shows the performance of FIT for ACRN according to the quarter. Owing to the low statistical power caused by the low proportion of ACRN, statistical differences in sensitivity were not identified. However, the sensitivity tended to be lower in the third and second quarters (16.1% [95% confidence interval [CI] 11.2–22.7%] and 18.0% [95% CI 12.2–25.6%], respectively) than in the fourth and first quarters (21.2% [95% CI 15.4–28.3%] and 22.2% [95% CI 16.2–29.7%], respectively). Specificity was higher in the second and third quarters (97.0% [95% CI 96.6–97.3%] and 96.8% [95% CI 96.5–97.2%], respectively) than in the fourth and first quarters (96.5% [95% CI, 96.1–96.8%] and 95.8% [95% CI 95.2–96.3%], respectively).

### Performance of fecal immunochemical test according to weather information

**Figure 3:**
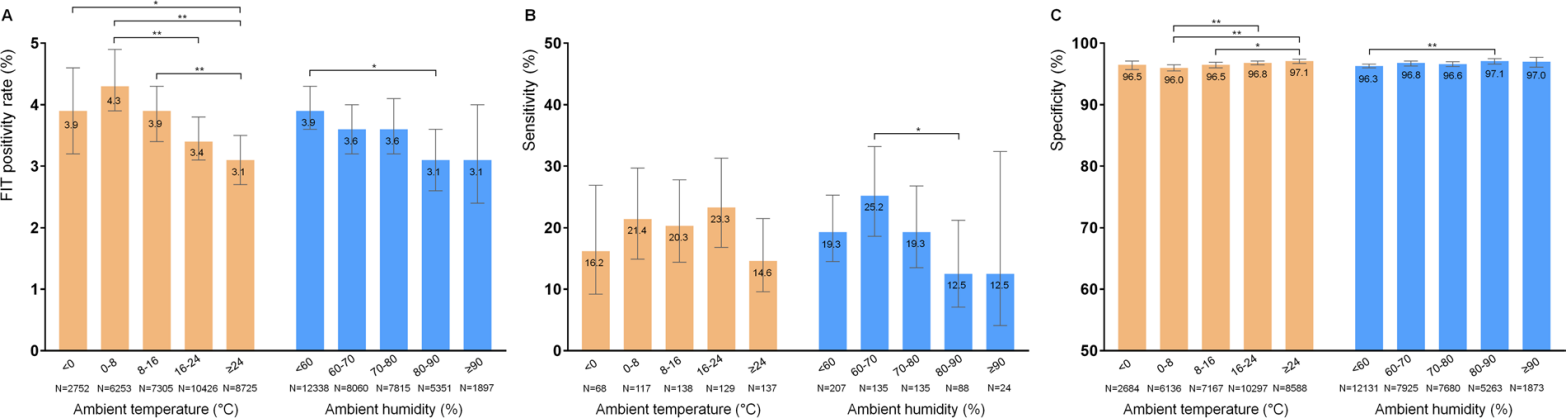
Diagnostic performance of fecal immunochemical test for advanced colorectal neoplasia according to different ambient temperatures and humidity. (**A**) positivity rate, (**B**) sensitivity, and (**C**) specificity of FIT. Bar represents 95% confidence interval. *P < 0.05, **P < 0.01, FIT, fecal immunochemical test.

The positivity rate, sensitivity, and specificity of FIT for ACRN were assessed according to ambient temperature and humidity (Fig. 3). The positivity rate of FIT was significantly lower at ≥24 °C than at <0 °C, 0–8 °C, and 8–16 °C; also, it was lower at 80–90% humidity than at <60% humidity.

Sensitivity tended to decrease at temperatures above 24 °C, although no statistical difference was identified. Moreover, sensitivity decreased at 80–90% humidity compared to 60–70% humidity. Although there was no statistical difference, ≥90% humidity tended to be related with low sensitivity.

In contrast, specificity increased at high ambient temperature and humidity. Specificity at an ambient temperature ≥24 °C was higher than at 0–8 °C or 8–16 °C. Specificity at 80–90% ambient humidity was higher than at <60% ambient humidity.

To assess the impact of ambient temperature and humidity on the sensitivity of FIT, we further performed a subgroup analysis of participants with CRC. Among 27 participants with CRC, 21 showed a positive result on FIT, and the sensitivity was 77.8%. The sensitivity was 100% in 3 participants who underwent FIT at a high ambient temperature (≥24 °C), while it was 50% in 4 participants who underwent FIT at high humidity (≥80%).

The results of subgroup analyses according to the type of FIT device (HM-JACK and OC-SENSOR DIANA) are shown in Table S1. Although statistical significance was identified only in a few comparisons due to the relatively small sample size in each subgroup, a decreasing tendency of sensitivity at high humidity was observed for both FIT devices.

### Logistic regression models for performance of fecal immunochemical test

Results of logistic regression analysis for the positivity rate, sensitivity, and specificity of FIT for ACRN are presented in Table 2. Based on the above results, high temperature was defined as ≥24 °C and high humidity as ≥80%. In the univariable model for positivity rate of FIT, both high ambient temperature and high ambient humidity were associated with a low positivity rate. Therefore, the interaction between high ambient temperature and high ambient humidity was also included in the multivariable analysis. High ambient temperature and humidity were associated with a low positivity rate of FIT (odds ratio [OR] 0.62, 95% CI 0.44–0.86). Neither high ambient temperature alone nor high ambient humidity alone was associated with the positivity rate of FIT in the multivariable analysis.

**Table 2:** Logistic regression models for diagnostic performance of fecal immunochemical test for advanced colorectal neoplasia.

Variable	Positivity rate					Sensitivity					Specificity				
	%	Crude OR (95% CI)	*P*-value	Adjusted OR (95% CI)	*P*-value	%	Crude OR (95% CI)	*P*-value	Adjusted OR (95% CI)	*P*-value	%	Crude OR (95% CI)	*P*-value	Adjusted OR (95% CI)	*P*-value
Age, /year	NA	1.02 (1.01–1.03)	<0.001	1.02 (1.01–1.03)	<0.001	NA	1.01 (0.99–1.03)	0.494	1.01 (0.98–1.03)	0.642	NA	0.98 (0.98–0.99)	<0.001	0.99 (0.98–0.99)	<0.001
**Sex**															
Male	3.5	0.93 (0.82–1.05)	0.245	0.95 (0.82–1.11)	0.538	19.7	1.12 (0.69–1.90)	0.654	1.13 (0.59–2.16)	0.709	96.8	1.12 (0.99–1.27)	0.080	1.08 (0.92–1.26)	0.337
Female	3.8	1		1		18.0	1		1		96.4	1		1	
**Family history of CRC**															
Absent	3.6	1		1		19.1	1		1		96.7	1		1	
Present	3.9	1.09 (0.81–1.43)	0.560	1.08 (0.80–1.42)	0.602	24.0	1.33 (0.48–3.24)	0.549	1.26 (0.45–3.08)	0.635	96.5	0.95 (0.71–1.30)	0.733	0.96 (0.72–1.32)	0.784
**Smoking history**															
Never smoker	3.6	1		1		18.3	1		1		96.6	1		1	
Former smoker	3.5	0.98 (0.85–1.13)	0.799	1.00 (0.86–1.18)	0.954	19.9	1.11(0.66–1.84)	0.695	1.07 (0.59–1.95)	0.827	96.8	1.06 (0.92–1.23)	0.422	1.02 (0.86–1.20)	0.839
Current smoker	3.8	1.06 (0.92–1.21)	0.420	1.14(0.97–1.33)	0.111	20.1	1.23 (0.69–1.83)	0.639	1.07 (0.59–1.95)	0.834	96.6	1.00 (0.87–1.15)	0.967	0.92 (0.78–1.08)	0.322
**Obesity**															
BMI < 25 kg/m^2^	3.7	1		1		20.3	1		1		96.6	1		1	
BMI ≥ 25 kg/m^2^	3.5	0.95 (0.85–1.07)	0.412	0.94 (0.84–1.06)	0.348	18.2	0.87 (0.57–1.32)	0.516	0.85 (0.56–1.29)	0.453	96.8	1.08 (0.95–1.22)	0.229	1.07 (0.95–1.22)	0.267
**Ambient temperature**															
<24 °C	3.8	1		1		20.8	1				96.5	1		1	
≥24 °C	3.1	0.80 (0.70–0.92)	0.002	0.96 (0.82–1.11)	0.563	14.6	0.70 (0.39–1.22)	0.110			97.1	1.17 (1.00–1.38)	0.006	1.02 (0.87–1.20)	0.855
**Ambient humidity**															
<80%	3.8	1		1		21.0	1		1		96.5	1		1	
≥80%	3.1	0.81 (0.70–0.94)	0.006	1.00 (0.84–1.18)	0.975	12.5	0.54 (0.28–0.95)	0.044	0.54 (0.28–0.96)	0.047	97.1	1.19 (1.02–1.39)	0.025	0.95 (0.80–1.14)	0.569
Interaction term temperature * humidity)															
≥24 °C and ≥80%				0.62(0.44–0.86)	0.005									1.80 (1.28–2.58)	<0.001

ACRN, advanced colorectal neoplasia; FIT, fecal immunochemical test; CRC, colorectal cancer; BMI, body mass index; OR, odds ratio; CI, confidence interval; NA, not applicable.

In the univariable analysis, high ambient humidity was associated with low sensitivity, but high ambient temperature was not. However, in the multivariable analysis, high ambient humidity was also associated with low sensitivity (OR 0.54, 95% CI 0.28–0.96).

In the univariable logistic regression model for specificity, both high ambient temperature and high ambient humidity were significant factors. In the multivariable analysis, high ambient temperature and humidity (temperature ≥24 °C and humidity ≥80%) were associated with high specificity (OR 1.80, 95% CI 1.28–2.58) of FIT. Neither high ambient temperature alone nor high ambient humidity alone was associated with specificity in the multivariable analysis.

### Changes in sensitivity and specificity according to cut-off values of fecal immunochemical test

**Figure 4:**
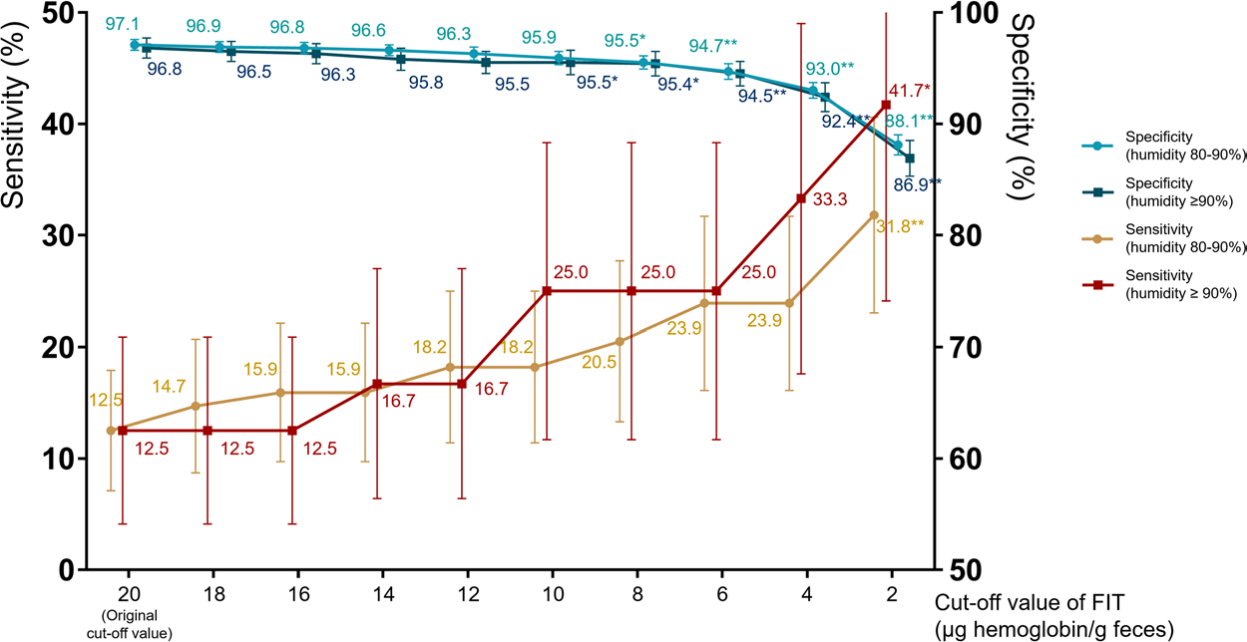
Changes in sensitivity and specificity of fecal immunochemical test for advanced colorectal neoplasia according to different cut-off values in high-humidity (≥80%) environments. Bar represents 95% confidence interval. Asterisk represents *P*-value of comparison between the reduced cut-off value and the original one (20 μg hemoglobin/g feces). *P < 0.05, **P < 0.01, FIT, fecal immunochemical test.

Figure 4 shows the changes in the sensitivity and specificity of FIT at 80–90% or ≥90% ambient humidity according to different cut-off values, which imply that environmental conditions resulted in the poor sensitivity. The sensitivity of FIT increased, whereas its specificity decreased with a decrease in cut-off values. Although no statistical significance was noted, the sensitivity tended to increase to over 20% when the cut-off value was reduced to 10 μg hemoglobin/g feces (a 50% reduction in the original cut-off value). When the cut-off value was assumed to be reduced by 50%, specificity decreased to 95.9% and 95.5% at 80–90% and ≥90% ambient humidity, respectively.

## Discussion

FIT is a widely accepted noninvasive tool for screening for CRC^6,20^. The sensitivity and specificity of FIT are unaffected by the characteristics of the individuals undergoing screening. Our previous study on the performance of FIT showed that the sensitivity was not affected by age, sex, smoking history, or obesity^12^. Although specificity decreased in individuals with diverticula or hemorrhoids, it was not influenced by clinical risk factors for ACRN, including male sex, obesity, and smoking^12^. Owing to these characteristics, FIT can be used for population-based screening regardless of the characteristics of the individuals being screened.

However, there is concern regarding the performance of FIT when fecal samples are obtained during the warm summer months^20^. Several studies have reported that the positivity rate of FIT was relatively low in the summer months^14–16^. In addition, a recent study by Cha *et al*. showed that the risk of interval cancer was 16% higher in individuals who underwent FIT in summer as opposed to in winter^13^.

Whether ambient temperature actually affects sensitivity and specificity remains unclear, because most previous studies evaluated the performance of FIT based on its positivity rate^14,15^. Although the sensitivity and specificity of FIT were assessed in the study by Cha *et al*., only individuals who were diagnosed with CRC within 1 year after undergoing FIT were regarded as true-positive cases^13^. Given that some of the individuals with false-negative results on FIT may not be diagnosed within 1 year, the sensitivity of FIT may have been overestimated in that study. In the current study, the sensitivity and specificity of FIT could be calculated accurately, because our cohort underwent both colonoscopy and FIT. In addition, our study showed that the sensitivity and specificity as well as the positivity rate of FIT were influenced by high ambient temperature and high humidity. The multivariable analysis demonstrated that high ambient temperature (≥24 °C) and humidity (≥80%) decreased the positivity rate and increased the specificity of FIT. In addition, high ambient humidity decreased the sensitivity of FIT. This is the first clinical study to show that high humidity and high temperature can reduce the quality of FIT. Our findings suggest that optimal humidity and temperature are important in maintaining the quality of FIT.

The impact of ambient temperature on the performance of FIT is supported by a previous experimental study by Vilkin *et al*.^21^, in which degradation of the fecal hemoglobin level was facilitated when samples were stored at 28 °C rather than at 4 °C and 20 °C. In addition, delayed return of fecal samples may cause false-negative results owing to hemoglobin degradation^22^. Another experimental study showed that minimizing both temperature and humidity is essential to maintain the integrity of the hemoglobin tetramer molecules^19^. When fecal samples were stored for 1 month at 37 °C, almost 100% of hemoglobin degraded at high humidity (>50%), whereas only 33–36% of hemoglobin degraded at low humidity (<30%)^19^. This result implies that not only temperature but also humidity is very important in maintaining the performance of FIT.

In addition, the impact of humidity on the performance of FIT may explain the contradictory results in previous studies focusing on the impact of ambient temperature on FIT. For example, Niedermaier *et al*. from Germany reported that the sensitivity and specificity of FIT did not differ between low-temperature and high-temperature groups^18^; this might be attributable to the different geographic areas, with different climate signatures, where the studies were conducted. In southern Germany, where the study by Niedermaier *et al*. was conducted, the summers are not very humid^23^. Relatively low humidity in summer might prevent deterioration of the sensitivity of FIT. In contrast to the study from Germany, a study performed in Florence, Italy and a nationwide Korean study showed seasonal variation in the diagnostic performance of FIT^13,14^, and Italy and Korea are not only very hot, but also very humid in summer^24,25^. In particular, Korea has a regional climate characteristic of the rainy season, and typhoons are frequent from July to September; therefore, there are many hot and humid days in summer. In summary, the diagnostic performance of FIT may be influenced by humidity as well as by temperature. This impact of humidity on the sensitivity of FIT seemed to be seen even in participants diagnosed with CRC. Although tests of statistical significance were not conducted due to the very small sample size, sensitivity for CRC tended to decrease at high humidity.

Considering that the hemoglobin level might be decreased on humid days, it may be reasonable to lower the cut-off values of FIT on such days. We therefore investigated the changes in the diagnostic performance of FIT by reducing the cut-off values in humid environments, and we found that the sensitivity of FIT increased from 12.5% to about 20% by reducing the cut-off value to 10 μg hemoglobin/g feces (a 50% reduction in the original cut-off value). However, at the same time, the specificity decreased from about 97% to 95.5–95.9%. Given that the specificity was 96.3–96.8% at low humidity, 95.5–95.9% specificity seemed to be relatively poor. Therefore, lowering the cut-off value for FIT performed on humid days is not the perfect solution. Performance of FIT should preferably be avoided on humid days, such as those in the rainy season in Korea. However, if carrying out the test is unavoidable on humid days, it should be kept in mind that the sensitivity of FIT may be decreased. In this situation, reinterpretation of the results of FIT based on the reduced cut-off value may be considered.

Although our study demonstrated the impact of ambient temperature and humidity on the performance of FIT for ACRN, it has several limitations. First, we could not investigate the return time of fecal samples, and it is well known that degradation of hemoglobin is influenced by time^19,21^. Van Rossum *et al*. reported that delays of more than 5 days in returning fecal samples increased false-negative results^22^. In our study, however, fecal samples were usually collected on the day before FIT was carried out or on the day of FIT. Second, two different devices were used for FIT, depending on the study period, and the performance of FIT may differ according to the type of device used. In the subgroup analyses, however, similar tendencies were observed in the diagnostic performance of FIT. In addition, there has been no evidence that the impact of seasonal variation differs according to the FIT device. The Korean study by Cha *et al*. showed that both types of tests (quantitative and qualitative FIT) had lower positivity rates in the summer months than in winter^13^. The type of buffer formulation used in the FIT may be an additional issue. Recently, a novel buffer formulation for the OC-Sensor FIT has been developed, to minimize hemoglobin degradation of fecal samples exposed to high temperatures. An *in vitro* study showed that the new formulation buffer enhanced hemoglobin-stabilizing properties in high temperature environments (>22 °C)^26^. However, the clinical implications of the use of this new buffer on CRC screening should be further evaluated in a subsequent study. Third, the number of participants with ACRN was relatively small, although our study included a large number of participants. Therefore, the statistical power in the analysis of sensitivity was relatively low, and we could not identify the impact of high ambient temperature on the sensitivity of FIT. Although ambient temperature was not independently associated with low sensitivity in our study, the influence of ambient temperature may not be completely ruled out, considering the results of previous studies. Larger studies may be required for reaching a definitive conclusion. Finally, this study was performed in Korea, which has a characteristic climate; therefore, it is difficult to generalize our findings worldwide. However, our results can be generalized to countries or regions with a climate similar to that of Korea.

Despite the limitations, our research enhances the understanding of the impact of weather parameters on the diagnostic performance of FIT. Ambient temperature and humidity may affect the performance of FIT for ACRN. High ambient humidity decreased the sensitivity of FIT. In addition, a high ambient temperature with high ambient humidity decreased the positivity rate of FIT. These results have important implications for FIT-based screening programs in countries where temperature and humidity vary widely by season. In countries with such a climate, FIT-based screening may have to be avoided during hot and humid seasons.

## Methods

The Kangbuk Samsung Health Study is a prospectively established cohort study of South Korean men and women aged ≥18 years, who underwent a comprehensive annual or biennial health examination at two large health checkup centers, namely the Kangbuk Samsung Hospital Total Healthcare Center in Seoul and in Suwon, South Korea. The current study participants are a subset of the population in the Kangbuk Samsung Health Study conducted between 2004 and 2015, and included participants who underwent both screening colonoscopy and FIT as part of a comprehensive health checkup.

Reasons for the large number of participants in this cohort who underwent both colonoscopy and FIT have been described previously^27^. In brief, according to the Industrial Safety and Health Law in Korea, employees should undergo a health checkup every one or two years. In addition, as part of the welfare policy, Korean companies often support the cost of health examinations, including CRC screening programs, regardless of the guidelines^27^. At our health checkup centers, a comprehensive health examination program includes screening colonoscopy and FIT. Although some participants chose either FIT or colonoscopy as a CRC screening modality, others prefer to undergo both examinations. The examinations carried out (namely FIT, colonoscopy, or both) depend on individual preference.

Among participants aged ≥30 years, those who met the following criteria were excluded: (i) previous colonic examination, colorectal surgery or CRN; (ii) history of inflammatory bowel disease; (iii) poor bowel preparation; and (iv) incomplete data for analysis. Poor bowel preparation was defined as “large amounts of solid fecal matter found, precluding a satisfactory study; unacceptable preparation; <90% visible mucosa”^28^.

This study was approved by the institutional review board of Kangbuk Samsung Hospital. The requirement for informed consent was waived since only de-identified data were retrospectively assessed. All experiments were performed in accordance with relevant guidelines and regulations.

### Measurements

Demographic data, including age, sex, height, weight, family history of CRC, smoking habits, underlying diseases, and medications, were retrieved from an electronic medical database. Data on family history of CRC, smoking habits, and underlying diseases had been collected based on a self-administered questionnaire method. A family history of CRC was defined as the presence of the disease in first-degree relatives at any age. Participants were considered as having a history of smoking if they were former or current smokers who had smoked ≥100 cigarettes in their life^29^. Body mass index (BMI) was calculated as measured weight (kg) divided by the square of the height (/m^2^), and obesity was defined as a BMI ≥ 25 kg/m^2^, which is the proposed cut-off value for Asian populations^30,31^.

### Colonoscopy and histopathological examination

Only board-certified endoscopists were involved in carrying out the colonoscopic examinations, which were performed using an EVIS LUCERA CV-260 colonoscope (Olympus Medical Systems, Tokyo, Japan). Four liters of polyethylene glycol solution were used for bowel cleansing. Suspicious neoplastic lesions were biopsied or removed by polypectomy or endoscopic mucosal resection.

CRN was defined as cancer or adenoma, and ACRN was defined as cancer or advanced adenoma. Advanced adenoma was defined as the presence of one of the following features: tumor diameter ≥10 mm, tubulovillous or villous structure, and high-grade dysplasia^32^.

### Fecal immunochemical test

A single fecal sample was collected at home, using a sampling tube containing buffer designed to minimize hemoglobin degradation, on the day before FIT was performed or on the day of FIT. After being sealed in a plastic bag, the fecal sample in the tube was hand-delivered to the laboratory on the day of FIT. Date of FIT was defined as the date on which the fecal sample arrived in the laboratory. The fecal hemoglobin level was determined using the HM-JACK test (Kyowa Medex Co. Ltd., Tokyo, Japan) from 2004 to 2009, and the OC-SENSOR DIANA test (Eiken Chemical Company, Tokyo, Japan) from 2010 to 2015. The cut-off value of FIT was 20 μg hemoglobin/g feces.

### Collection of weather information

Data about daily air temperature and relative humidity during the study period were collected from the official website of the Korea Meteorological Administration (www.weather.go.kr/weather/climate/past_table.jsp). As two centers, one in Seoul and the other in Suwon, were included in this study, weather information was collected for each of these locations. In this study, ambient temperature was defined as the average temperature of the day. The daily weather information was then merged into the clinical dataset of each participant, based on the date of FIT.

### Statistical analysis

Continuous variables were presented as mean with standard deviation. Categorical variables were presented as numbers and proportions. The chi-square test was used for group comparisons.

Temperature and humidity were divided into five levels: <0 °C, 0–8 °C, 8–16 °C, 16–24 °C, and ≥24 °C for temperature; and <60%, 60–70%, 70–80%, 80–90%, and ≥90% for humidity. The positivity rate, sensitivity, and specificity of FIT for ACRN were calculated according to ambient temperature and humidity. Subgroup analyses were performed according to the type of FIT device used, to assess the impact of the device used on the diagnostic performance of FIT.

Logistic regression analysis was conducted to evaluate the impact of high temperature or high humidity on the positivity rate, sensitivity, and specificity of FIT. Here the well-known risk factors of ACRN, including age, sex, family history of CRC, smoking history, and obesity, were adjusted as confounding variables. With regard to temperature and humidity, only variables that showed statistical significance in the univariable analysis were included in the multivariable analysis. If both temperature and humidity were included in the multivariable analysis, the interaction term (temperature * humidity) was also included in the model, in order to consider the rainy season in Korea (usually in July and August), with high temperatures and high humidity.

Finally, to evaluate whether poor sensitivity of FIT in summer (or on hot and humid days) would improve by adjusting the cut-off value of FIT, we assessed changes in sensitivity and specificity when the cut-off value was reduced.

A *P-*value < 0.05 was regarded as being significant in group comparisons. Statistical analyses were performed using statistical software R (version 3.5.2; R Foundation for Statistical Computing, Vienna, Austria).

## Supplementary information

Table S1. Diagnostic performance of the fecal immunochemical test according to the type of device used, ambient temperature, and ambient humidity.
