# Supplementary material for: Impact of temperature and humidity on performance of the fecal immunochemical test for advanced colorectal neoplasia
Source: Sci Rep. 2019 Jul 8;9:9824. doi: 10.1038/s41598-019-44490-y (PMC6614420; doi:10.1038/s41598-019-44490-y)
Supplement: Supplementary file 1 — Table S1. Diagnostic performance of the fecal immunochemical test according to the type of device used, ambient temperature, and ambient humidity. [file 41598_2019_44490_MOESM1_ESM.docx]

**Impact of temperature and humidity on performance of the fecal immunochemical test for advanced colorectal neoplasia**

**Chan Hyuk Park^1^, Yoon Suk Jung^2^, Nam Hee Kim^3^, Mi Yeon Lee^4^, Jung Ho Park^5^, Dong Il Park^6^, and Chong Il Sohn^7^**

^1^Department of Internal Medicine, Hanyang University Guri Hospital, Hanyang University College of Medicine, Guri, Korea

^2^Division of Gastroenterology, Department of Internal Medicine, Kangbuk Samsung Hospital, Sungkyunkwan University School of Medicine, Seoul, Korea

^3^Preventive Health Care, Kangbuk Samsung Hospital, Sungkyunkwan University School of Medicine, Seoul, Korea

^4^Division of Biostatistics, Department of R&D Management, Kangbuk Samsung Hospital, Sungkyunkwan University School of Medicine, Seoul, Republic of Korea

^5^Division of Gastroenterology, Department of Internal Medicine, Kangbuk Samsung Hospital, Sungkyunkwan University School of Medicine, Seoul, Korea

^6^Division of Gastroenterology, Department of Internal Medicine, Kangbuk Samsung Hospital, Sungkyunkwan University School of Medicine, Seoul, Korea

^7^Division of Gastroenterology, Department of Internal Medicine, Kangbuk Samsung Hospital, Sungkyunkwan University School of Medicine, Seoul, Korea

**Correspondence to:** Yoon Suk Jung, MD, PhD

Division of Gastroenterology, Department of Internal Medicine, Kangbuk Samsung Hospital, Sungkyunkwan University School of Medicine, 29, Saemunan-Ro, Jongno-Gu, Seoul 03181, Korea

Phone: +82-2-2001-8577, Fax: +82-2-2001-2049

E-mail: ys810.jung@samsung.com

| Table S1. Diagnostic performance of the fecal immunochemical test according to the type of device used, ambient temperature, and ambient humidity. | | | | | | | | | | | | | | | | |
| --- | --- | --- | --- | --- | --- | --- | --- | --- | --- | --- | --- | --- | --- | --- | --- | --- |
| Type of FIT device | Variable | | FIT positive rate | | | |  | Sensitivity | | | |  | Specificity | | | |
|  |  |  | FIT positive, n | N | % (95% CI) | *P*-value |  | ACRN detected by FIT, n | ACRN, n | % (95% CI) | *P*-value |  | No ACRN by FIT, n | No ACRN, n | % (95% CI) | *P*-value |
| HM-JACK test | Ambient temperature, °C | |  |  |  |  |  |  |  |  |  |  |  |  |  |  |
|  |  | < 0 | 52 | 1118 | 4.7 (3.6-6.1) | 0.273 |  | 4 | 20 | 20.0 (7.7-42.8) | >0.999 |  | 1050 | 1098 | 95.6 (94.2-96.7) | 0.261 |
|  |  | 0 - 8 | 126 | 2273 | 5.5 (4.7-6.6) | Reference |  | 8 | 35 | 22.9 (11.9-39.5) | Reference |  | 2120 | 2238 | 94.7 (93.7-95.6) | Reference |
|  |  | 8 - 16 | 104 | 1962 | 5.3 (4.4-6.4) | 0.728 |  | 7 | 36 | 19.4 (9.6-35.5) | 0.725 |  | 1829 | 1926 | 95.0 (93.9-95.9) | 0.731 |
|  |  | 16 - 24 | 135 | 2802 | 4.8 (4.1-5.7) | 0.245 |  | 12 | 43 | 27.9 (16.6-43.0) | 0.611 |  | 2636 | 2759 | 95.5 (94.7-96.3) | 0.181 |
|  |  | ≥24 | 78 | 1709 | 4.6 (3.7-5.7) | 0.165 |  | 0 | 19 | 0.0 (0.0-29.8) | 0.040 |  | 1612 | 1690 | 95.4 (94.3-96.3) | 0.349 |
|  | Ambient humidity, % | |  |  |  |  |  |  |  |  |  |  |  |  |  |  |
|  |  | <60 | 241 | 4269 | 5.6 (5.0-6.4) | Reference |  | 11 | 65 | 16.9 (9.6-28.0) | 0.133 |  | 3974 | 4204 | 94.5 (93.8-95.2) | Reference |
|  |  | 60 - 70 | 111 | 2554 | 4.3 (3.6-5.2) | 0.019 |  | 12 | 41 | 29.3 (17.4-44.8) | Reference |  | 2414 | 2513 | 96.1 (95.2-96.8) | 0.005 |
|  |  | 70 - 80 | 98 | 1903 | 5.1 (4.2-6.2) | 0.430 |  | 7 | 27 | 25.9 (12.9-45.3) | 0.764 |  | 1785 | 1876 | 95.1 (94.1-96.0) | 0.318 |
|  |  | 80 - 90 | 35 | 934 | 3.7 (2.7-5.2) | 0.019 |  | 1 | 18 | 5.6 (0.8-30.7) | 0.049 |  | 882 | 916 | 96.3 (94.8-97.3) | 0.029 |
|  |  | ≥90 | 10 | 204 | 4.9 (2.7-8.9) | 0.652 |  | 0 | 2 | 0.0 (0.0-80.6) | >0.999 |  | 192 | 202 | 95.0 (91.0-97.3) | 0.750 |
| OC-SENSOR DIANA test | Ambient temperature, °C | |  |  |  |  |  |  |  |  |  |  |  |  |  |  |
|  |  | < 0 | 54 | 1634 | 3.3 (2.5-4.3) | 0.533 |  | 7 | 48 | 14.6 (7.1-27.6) | 0.383 |  | 1539 | 1586 | 97.0 (96.1-97.8) | 0.541 |
|  |  | 0 - 8 | 145 | 3980 | 3.6 (3.1-4.3) | Reference |  | 17 | 82 | 20.7 (13.3-30.8) | Reference |  | 3770 | 3898 | 96.7 (96.1-97.2) | Reference |
|  |  | 8 - 16 | 178 | 5343 | 3.3 (2.9-3.8) | 0.416 |  | 21 | 102 | 20.6 (13.8-29.5) | 0.981 |  | 5084 | 5241 | 97.0 (96.5-97.4) | 0.433 |
|  |  | 16 - 24 | 222 | 7624 | 2.9 (2.6-3.3) | 0.033 |  | 18 | 86 | 20.9 (13.6-30.8) | 0.975 |  | 7334 | 7538 | 97.3 (96.9-97.6) | 0.081 |
|  |  | ≥24 | 190 | 7016 | 2.7 (2.4-3.1) | 0.006 |  | 20 | 118 | 16.9 (11.2-24.8) | 0.498 |  | 6728 | 6898 | 97.5 (97.1-97.9) | 0.013 |
|  | Ambient humidity, % | |  |  |  |  |  |  |  |  |  |  |  |  |  |  |
|  |  | <60 | 245 | 8069 | 3.0 (2.7-3.4) | 0.444 |  | 29 | 142 | 20.4 (14.6-27.8) | 0.586 |  | 7711 | 7927 | 97.3 (96.9-97.6) | Reference |
|  |  | 60 - 70 | 180 | 5506 | 3.3 (2.8-3.8) | Reference |  | 22 | 94 | 23.4 (15.9-33.0) | Reference |  | 5254 | 5412 | 97.1 (96.6-97.5) | 0.504 |
|  |  | 70 - 80 | 186 | 5912 | 3.1 (2.7-3.6) | 0.709 |  | 19 | 108 | 17.6 (11.5-25.9) | 0.306 |  | 5637 | 5804 | 97.1 (96.7-97.5) | 0.592 |
|  |  | 80 - 90 | 129 | 4417 | 2.9 (2.5-3.5) | 0.320 |  | 10 | 70 | 14.3 (7.9-24.6) | 0.145 |  | 4228 | 4347 | 97.3 (96.7-97.7) | 0.967 |
|  |  | ≥90 | 49 | 1693 | 2.9 (2.2-3.8) | 0.442 |  | 3 | 22 | 13.6 (4.5-34.8) | 0.399 |  | 1625 | 1671 | 97.2 (96.3-97.9) | 0.949 |
| ACRN, advanced colorectal neoplasia; FIT, fecal immunochemical test; CI, confidence interval | | | | | | | | | | | | | | | | |
